# Extensor Pollicis Longus Laceration

**Published:** 2013-01-15

**Authors:** Izabela Galdyn, Janet H. Yueh, Mark S. Granick

**Affiliations:** ^a^Northeast Ohio Medical University, Rootstown; ^b^Division of Plastic Surgery, University of Medicine and Dentistry of New Jersey, Newark

**Figure:**
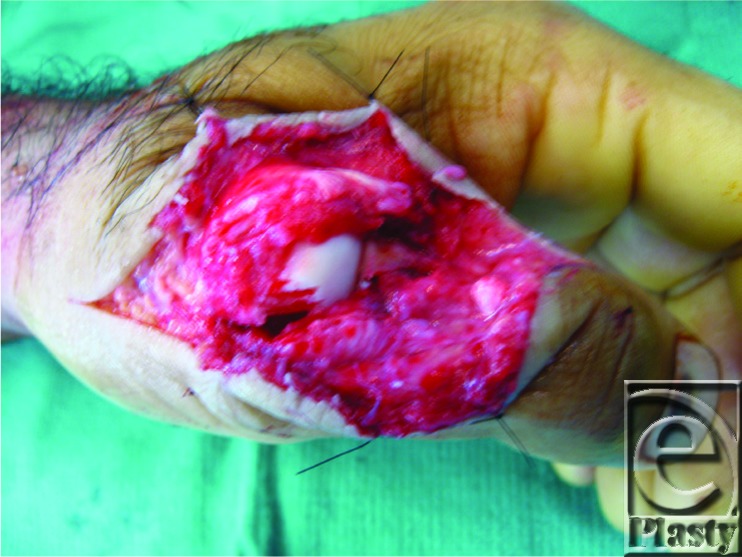


## DESCRIPTION

A 35-year-old man suffered a self-inflicted work injury from a circular saw on the dorsum of the thumb on his nondominant hand, sustaining a total transection of his extensor pollicus longus (EPL) and a concomitant dorsal capsule injury.

## QUESTIONS

1. **Discuss initial workup including findings of physical examination and imaging.**
2. **Where does the EPL retract after laceration injuries?**
3. **How does one repair a concomitant capsular injury?**
4. **What is the appropriate splinting regimen after EPL repair?**

## DISCUSSION

Table saws are a common cause of finger lacerations particularly in the working population. When confronted with this type of injury, it is important to assess the circulation of the affected digit by checking for appropriate capillary refill as well as digit color and warmth, the range of motion to verify tendon injury, and the innervation as tested by light sensation and 2-point discrimination. It is important to understand the blood supply to the thumb to determine full extent of any injury. There are 2 sets of digital vessels, which supply the thumb, a radial and ulnar dorsal pair and an ulnar and radial digital pair, which run volarly. Depending on the trajectory of the saw, one or more of these vessels could be lacerated, which will be important in determining the course of any subsequent repair.^1^ Once a better understanding of the precise deficits has been obtained, a proper course of treatment can be determined.

**Figure 1:**
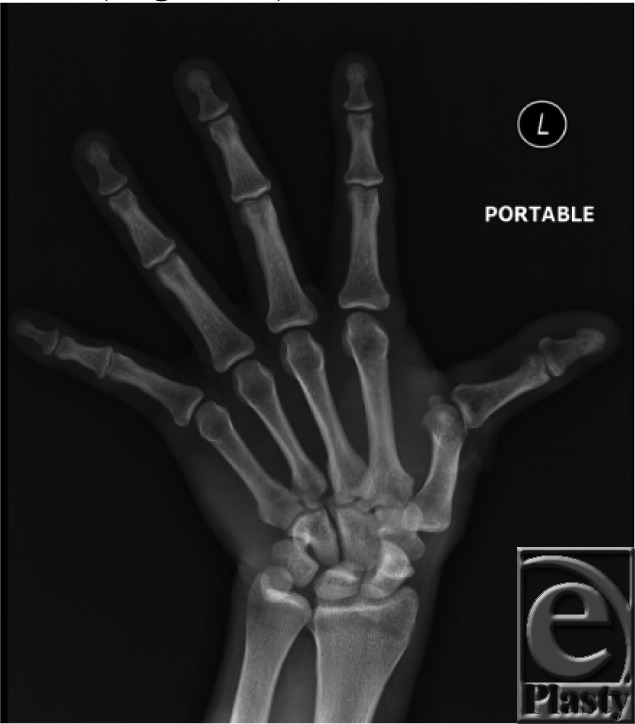
Radial deviation of the thumb is typical with a laceration of the extensor pollicis longus.

In addition to having an IP joint extensor deficit, our patient also presented with a significantly radially deviated thumb (Fig 1). This positioning of the thumb alerted us to a possible injury of the ulnar collateral ligament. However, our initial examination was limited due to significant pain, and we were unable to adequately assess this injury until we were in the operating room. To properly test for ulnar collateral instability, a valgus stress test is performed at 0° and 30° of flexion showing greater than 30° and 15° of movement, respectively. Often these injuries are associated with dorsal capsular tears.^2^ Once the patient was sedated, a proper examination was performed and no valgus laxity was noted and the collateral ligament was determined to be intact. As shown in the cover image, the patient clearly had a coexistent dorsal capsular tear with an exposed metacarpal head. Without repairing the capsule, the newly repaired EPL would sublux ulnarly and volarly, leading to a significant extensor lag.^3^ The recommendation is to repair the capsule separately from the EPL tendon that was performed using a 4-0 Ethibond in a horizontal mattress fashion.

Once a tendon is lacerated, it has a tendency to retract, making location of the proximal end difficult. Given a particular point of laceration and knowledge of the EPL attachments, the position of the lacerated tendon can be determined. If the EPL is transected distally to the MCP, the sagittal fibers from the abductor pollicis brevis and adductor pollicis will keep it from retracting. If transected proximally to the MCP, the tendon retracts to the proximal carpal row where it is held by synovial sheet communications from the extensor carpi radialis tendons.^5^^-^6

We repaired the EPL tendon first by debriding the lacerated edges and then by performing a Strickland repair using 4-0 nonabsorbable polyester suture. The patient was placed in a thumb-spica splint postoperatively. The recommendations for splinting are varied, with standard evidence saying there is no difference between static and dynamic splinting in terms of return to function and range of motion.^4^ However, more recent studies have shown that dynamic splinting within 7 days of repair with continuation for 3 weeks favors greater tendon excursion and leads to a lower rate of adhesions.^7^

## References

[1] Ramirez AR, Gonzalez SM (2012). Arteries of the thumb: description of anatomical variations and review of literature. Plast Reconstr Surg.

[2] Chuter GSJ, Muwanga CL, Irwin LR (2009). Ulnar collateral ligament injuries of the thumb: 10 years of surgical experience. Injury.

[3] Krause JO, Manske PR, Mirly HL, Szerzinski J (1996). Isolated injuries of the dorsoradial capsule of the thumb metacarpophalangeal joint. J Hand Surg Am.

[4] Soni P, Stern CA, Foreman KB, Rockwell WB (2009). Advances in extensor tendon diagnosis and therapy. Plast Reconstr Surg.

[5] Jaibaji M, Rayan GM, Chung KW (2008). Functional anatomy of the thumb sagittal band. J Hand Surg Am.

[6] De Maeseneer M, Marcelis S, Osteaux M, Jager T, Machiels F, Van Roy P (2005). Sonography of a rupture of the tendon of the extensor pollicis longus muscle: initial clinical experience and correlation with findings at cadaveric dissection. AJR Am J Roentgenol.

[7] Neuhaus V, Wong G, Russo KE, Mugdal CS (2012). Dynamic splinting with early motion following zone IV/V and TI to TIII extensor tendon repairs. J Hand Surg Am.

